# The Effective Coverage of Homogeneous Teams with Radial Attenuation Models

**DOI:** 10.3390/s23010350

**Published:** 2022-12-29

**Authors:** Yuan-Rui Yang, Qiyu Kang, Rui She

**Affiliations:** 1 School of Mechanical and Aerospace Engineering, Nanyang Technological University, Singapore 637460, Singapore; 2 Centre for Information Sciences and Systems, Nanyang Technological University, Singapore 639798, Singapore

**Keywords:** coverage control, multi-robot, cooperative sensors, optimization

## Abstract

For the area coverage (e.g., using a WSN), despite the comprehensive research works on full-plane coverage using a multi-node team equipped with the ideal constant model, only very few works have discussed the coverage of practical models with varying intensity. This paper analyzes the properties of the effective coverage of multi-node teams consisting of a given numbers of nodes. Each node is equipped with a radial attenuation disk model as its individual model of coverage, which conforms to the natural characteristics of devices in the real world. Based on our previous analysis of 2-node teams, the properties of the effective coverage of 3-node and *n*-node (n≥4) teams in regular geometric formations are analyzed as generalized cases. Numerical analysis and simulations for 3-node and *n*-node teams (n≥4) are conducted separately. For the 3-node cases, the relations between the side lengths of equilateral triangle formation and the effective coverage of the team equipped with two different types of models are respectively inspected. For the *n*-node cases (n≥4), the effective coverage of a team in three formations, namely regular polygon, regular star, and equilateral triangular tessellation (for n=6), are investigated. The results can be applied to many scenarios, either dynamic (e.g., robots with sensors) or static, where a team of multiple nodes cooperate to produce a larger effective coverage.

## 1. Introduction

### 1.1. Background

Many static devices and autonomous robots (collectively named “nodes”) can collaboratively sense or influence their surroundings. Coverage (area coverage) and effective coverage [1] are important metrics that reflect the sensing or influencing ability, or the performance of a node or a node team, such as a light array, air-conditioner team, wireless sensor network, etc. The analysis of a team’s effective coverage depends on the problems to be solved and their adopted models of coverage. The most common categories of problems—optimal tessellation patterns (e.g., sensor placement issue) in a given plane have been extensively studied. Most of them have adopted the simplest binary disk model (having a constant value within the disk region centered at the node), which focuses on *k*-coverage [2,3]; only a very few have analyzed the effective coverage of cooperative sensors within an infinite plane using a non-binary (non-constant) sensing model [4,5].

Compared to this category of problems, the optimization of the effective coverage of a node team containing a given number of nodes in a certain operational setting (i.e., the inverse problem) has been rarely studied due to its difficulties in mathematics. For practical models with non-constant intensities (i.e., non-constant models), both maximizing the team’s effective coverage and maintaining its connectedness are much more complicated than the ideal binary model [6,7] with which the nodes may move freely to independently produce some effective coverage. If the coverage intensity of one node is already larger than the requirement, the team can achieve its maximum coverage by simply avoiding any overlap among the individual coverage disks. However, for non-constant models, the area and the connectedness of a team’s effective coverage have complicated relationships with the team’s formation; any change in the team’s formation causes changes in its effective coverage. However, it is useful in and can be applied to many dynamic problems involving moving robots, such as mobile WSNs, cooperative illumination, animal husbandry, and agriculture (for birds detection/driving), etc. [1,8]. Hence, maximizing the effective coverage of a node team and maintaining the connectedness of its effectively covered region are desirable in such practical applications, which motivates our study in this article.

### 1.2. Literature Review

The optimal tessellation pattern problem (to fully cover a plane) using the binary disk model (a constant non-zero intensity within the coverage disk) has been extensively studied (e.g., [2,3,9,10,11,12,13,14]) based on the binary disk coverage model. Many other works involving multi-node cooperative coverage (e.g., [15,16,17]) also adopt this model. However, such a model is too simple to characterize most realistic devices.

Some new non-constant models (with varying intensity) have been proposed [4,5,7,18] for sensing activities, where multiple sensors are cooperatively used to enlarge the sensing area and to improve the sensing accuracy. For example, in [5], Zhu et al. proposed that a point is “confident information covered” if the root-mean-square error of its estimated signal value is below the application requirement. Using attenuation sensing models, cooperative sensors can produce a larger “confident information coverage” than the sum of individual sensor coverages. These works found the optimal placement pattern (with the lowest sensor density) for an infinite plane under their model assumption. This model was also adopted in several later works [19,20]. Yáng et al. proposed the concept of effective coverage based on the coverage intensity to characterize the coverage behavior of practical devices equipped with attenuation models [1]. Their model follows the superposition principle, which focuses on influencing activities (output coverage) and can approximate the behavior of sensing activities. Effective coverages of 2-node teams equipped with different models were analyzed. Meng et al. proposed a learning method for the effective coverage of points of interest, where the sensing quality of an agent is a decreasing function of the distance from this agent [21].

In this paper, we study the effective coverage of *n*-node homogeneous teams with n≥3. The nodes are equipped with radial attenuation disk models to approximate realistic devices. A team’s (cooperative) coverage follows the superposition principle. Optimal separation distances that maximize a team’s effective coverage are analyzed and simulated, respectively, for two types of models of coverage. The results can be directly applied to practical applications such as mobile air-conditioner teams [22] and birds driving robots, for output applications [1]; and for input applications, e.g., mobile wireless sensor networks, the approximation using our models and analysis is much more accurate and practical than the existing binary models. To the best of our knowledge, this is the first work to study the effective coverage optimization of a team containing a given number (≥3) of nodes equipped with a non-constant model of coverage.

The rest of the paper is organized as follows. Section 2 formulates our model and then analyzes 3-node teams and *n*-node teams, respectively. The analysis and simulation of some example models are presented in Section 3. Finally, we conclude in Section 4.

## 2. Coverage of an n-Node Team in Regular Polygon Formation

The situation of an *n*-node team with n≥3 is much more complicated compared to 2-node teams. It is hard to find their explicit analytical relationship (function) with the team’s effective coverage, and there are infinite possibilities of formations on a 2D plane. Therefore, for n>3, only some symmetric cases are analyzed, where the teams are in regular polygon and regular star formations.

**Figure 1:**
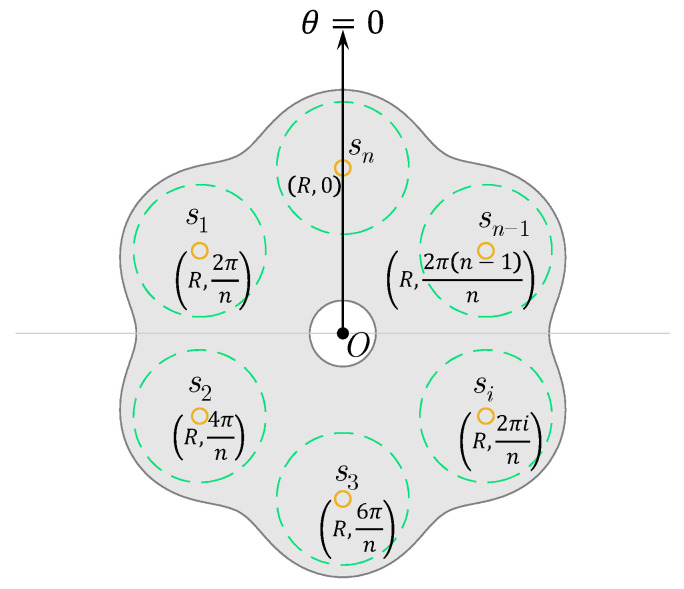
Illustration of an *n*-node team forming an *n*-sided regular polygon, with the nodes being located on its vertices. The shaded gray region is the effectively covered region (an example) by the team, while the *n* dashed circles show individual effective coverages with radius $\bar{r}$. The polar coordinate system is established in such a way where the pole is located at the circumcenter of the polygon, and the polar axis points towards $s_{n}$.

We consider a set of *n* nodes located at planar positions $s_{i}$ with i=1,2,…,n. As the vertices of a polygon, they are in the formation of an *n*-sided regular polygon with a circumradius *R*. This set of nodes can be represented as a network $S=\{s_{1},\ldots ,s_{n}\}$. We establish a polar coordinate system so that the pole is at the circumcenter *O* of the *n*-sided regular polygon, and the polar axis crosses the node $s_{n}$. We use *D* to represent the side length of the polygon formation, i.e., the separation of any two adjacent nodes on the polygon vertices. So, $s_{i}=\left(R,\frac{2\pi i}{n}\right)$ and R=0.5Dcsc(π/n), as illustrated in Figure 1. The distance between any point $p\left(\rho \geq 0,\theta \right)$ and node $s_{i}$ located at the polygon vertex $\left(R,\frac{2\pi i}{n}\right)$ is then $d_{i}=\sqrt{R^{2}+\rho^{2}-2R\rho \cos\left(\theta -\frac{2\pi i}{n}\right)}$.

The coverage intensity induced by a node $s_{i}\in S$ at a point $p\in R^{2}$ is represented as a non-constant function $I(d_{i}):R\to R$, where $d_{i}=∥p-s_{i}∥$ is the distance from $s_{i}$ to *p*. The coverage of a node $s_{i}$ (denoted by $C_{i}$) is $C_{i}={\;\int \int \;}_{R^{2}}\delta_{i}\left(p\right)\;dp$, where $\delta_{i}\left(p\right)=1$ if $I(d_{i})>0$ and $\delta_{i}\left(p\right)=0$ otherwise. The individual coverage radius is denoted by $r\in R^{+}$. With a certain threshold $\gamma \in R^{+}$, the effective coverage of a node $s_{i}$ (denoted by ${\bar{C}}_{i}$) is ${\bar{C}}_{i}={\;\int \int \;}_{R^{2}}{\bar{\delta }}_{i}\left(p\right)\;dp$, where ${\bar{\delta }}_{i}\left(p\right)=1$ if $I(d_{i})\geq \gamma$, and ${\bar{\delta }}_{i}\left(p\right)=0$ otherwise. From this definition, we know that ${\bar{C}}_{S}={\;\int \int \;}_{R^{2}}\bar{\delta }\left(p\right)\;dp$, where $\bar{\delta }\left(p\right)=1$ if Φ(p)≥γ and $\bar{\delta }\left(p\right)=0$ otherwise. For one team, there are two critical separation distances:

1. “Max distance”, which yields the maximum team’s effective coverage.
2. “Last-connection distance”. Any separation *D* greater than this distance will make the team’s effectively covered region disconnect into more than one part.

### 2.1. Three-Node Teams

For a three-node team forming an equilateral triangle, the nodes are located at the polar points $\left(R,2\pi /3\right)$, $\left(R,-2\pi /3\right)$, $\left(R,0\right)$. The coverage intensity at any point p(ρ≥0,θ) induced by the team is

(1) $$\begin{array}{cc} \Phi (p)= & I\left(\sqrt{R^{2}+\rho^{2}-2R\rho \cos\left(\theta -\frac{2}{3}\pi \right)}\right)\\ & +I\left(\sqrt{R^{2}+\rho^{2}-2R\rho \cos\left(\theta +\frac{2}{3}\pi \right)}\right)+I\left(\sqrt{R^{2}+\rho^{2}-2R\rho \cos\theta }\right) \end{array}$$

and its first-order and second-order partial derivative w.r.t. ρ are

(2) $$\begin{array}{cc} \frac{\partial \Phi (p)}{\partial \rho }= & {\left.\frac{dI}{dd}\right|}_{d=\sqrt{R^{2}+\rho^{2}-2R\rho \cos\left(\theta -\frac{2}{3}\pi \right)}}\frac{\rho -R\cos\left(\theta -\frac{2}{3}\pi \right)}{\sqrt{R^{2}+\rho^{2}-2R\rho \cos\left(\theta -\frac{2}{3}\pi \right)}}\\ \; & +{\left.\frac{dI}{dd}\right|}_{d=\sqrt{R^{2}+\rho^{2}-2R\rho \cos\left(\theta +\frac{2}{3}\pi \right)}}\frac{\rho -R\cos\left(\theta +\frac{2}{3}\pi \right)}{\sqrt{R^{2}+\rho^{2}-2R\rho \cos\left(\theta +\frac{2}{3}\pi \right)}}\\ \; & +{\left.\frac{dI}{dd}\right|}_{d=\sqrt{R^{2}+\rho^{2}-2R\rho \cos\theta }}\frac{\rho -R\cos\theta }{\sqrt{R^{2}+\rho^{2}-2R\rho \cos\theta }}, \end{array}$$

(3) $$\begin{array}{cc} \; & \frac{\partial^{2}\Phi \left(p\right)}{\partial \rho^{2}}=\\ \; & {\left.\frac{d^{2}I\left(d\right)}{dd^{2}}\right|}_{d=d_{1}}\frac{{\left[\rho -R\cos\left(\theta -\frac{2\pi }{3}\right)\right]}^{2}}{R^{2}+\rho^{2}-2R\rho \cos\left(\theta -\frac{2\pi }{3}\right)}+{\left.\frac{dI(d)}{dd}\right|}_{d=d_{1}}\frac{R^{2}\sin^{2}\left(\theta -\frac{2\pi }{3}\right)}{{\left[R^{2}+\rho^{2}-2R\rho \cos\left(\theta -\frac{2\pi }{3}\right)\right]}^{1.5}}\\ + & {\left.\frac{d^{2}I\left(d\right)}{dd^{2}}\right|}_{d=d_{2}}\frac{{\left[\rho -R\cos\left(\theta +\frac{2\pi }{3}\right)\right]}^{2}}{R^{2}+\rho^{2}-2R\rho \cos\left(\theta +\frac{2\pi }{3}\right)}+{\left.\frac{dI(d)}{dd}\right|}_{d=d_{2}}\frac{R^{2}\sin^{2}\left(\theta +\frac{2\pi }{3}\right)}{{\left[R^{2}+\rho^{2}-2R\rho \cos\left(\theta +\frac{2\pi }{3}\right)\right]}^{1.5}}\\ + & {\left.\frac{d^{2}I\left(d\right)}{dd^{2}}\right|}_{d=d_{3}}\frac{{\left[\rho -R\cos\theta \right]}^{2}}{R^{2}+\rho^{2}-2R\rho \cos\theta }+{\left.\frac{dI(d)}{dd}\right|}_{d=d_{3}}\frac{R^{2}\sin^{2}\theta }{{\left[R^{2}+\rho^{2}-2R\rho \cos\theta \right]}^{1.5}} \end{array}$$

where $d_{1}=\sqrt{R^{2}+\rho^{2}-2R\rho \cos\left(\theta -\frac{2}{3}\pi \right)}$, $d_{2}=\sqrt{R^{2}+\rho^{2}-2R\rho \cos\left(\theta +\frac{2}{3}\pi \right)}$, and $d_{3}=\sqrt{R^{2}+\rho^{2}-2R\rho \cos\theta }$. At the circumcenter O(0,0),

(4) Φ(O)=3I(R)

(5) $${\left.\frac{\partial \Phi (p)}{\partial \rho }\right|}_{\rho =0}=-{\left.\frac{dI}{dd}\right|}_{d=R}\left[\cos\left(\theta -\frac{2}{3}\pi \right)+\cos\left(\theta +\frac{2}{3}\pi \right)+\cos\theta \right]=0$$

(6) $$\begin{array}{cc} {\left.\frac{\partial^{2}\Phi \left(p\right)}{\partial \rho^{2}}\right|}_{\rho =0}= & {\left.\frac{d^{2}I\left(d\right)}{dd^{2}}\right|}_{d=R}\cos^{2}\left(\theta -\frac{2}{3}\pi \right)+{\left.\frac{dI(d)}{dd}\right|}_{d=R}\frac{\sin^{2}\left(\theta -\frac{2}{3}\pi \right)}{R}\\ \; & +{\left.\frac{d^{2}I\left(d\right)}{dd^{2}}\right|}_{d=R}\cos^{2}\left(\theta +\frac{2}{3}\pi \right)+{\left.\frac{dI(d)}{dd}\right|}_{d=R}\frac{\sin^{2}\left(\theta +\frac{2}{3}\pi \right)}{R}\\ \; & +{\left.\frac{d^{2}I\left(d\right)}{dd^{2}}\right|}_{d=R}\cos^{2}\theta +{\left.\frac{dI(d)}{dd}\right|}_{d=R}\frac{\sin^{2}\theta }{R}\\ = & {\left.\frac{d^{2}I\left(d\right)}{dd^{2}}\right|}_{d=R}\frac{3+\cos\left(2\theta -\frac{4}{3}\pi \right)+\cos\left(2\theta +\frac{4}{3}\pi \right)+\cos2\theta }{2}\\ \; & +{\left.\frac{dI(d)}{dd}\right|}_{d=R}\frac{3-\cos\left(2\theta -\frac{4}{3}\pi \right)-\cos\left(2\theta +\frac{4}{3}\pi \right)-\cos2\theta }{2R}\\ = & {\left.\frac{d^{2}I\left(d\right)}{dd^{2}}\right|}_{d=R}\frac{3}{2}+{\left.\frac{dI(d)}{dd}\right|}_{d=R}\frac{3}{2R}\\ = & \frac{3}{2}\left[{\left.\frac{d^{2}I\left(d\right)}{dd^{2}}\right|}_{d=R}+{\left.\frac{dI(d)}{dd}\right|}_{d=R}\frac{1}{R}\right] \end{array}$$

Obviously, if I(d) is a concave model, dI(d)/dd<0 and $d^{2}I\left(d\right)/dd^{2}<0$, and Equation (6) is negative; otherwise if I(d) is a convex model, Equation (6) is positive; see Appendix B for the proof. Hence, the circumcenter *O* is a local minimum point of coverage intensity when the nodes are equipped with a convex model of coverage; and it is a local maximum point of coverage intensity when the nodes are equipped with a concave model of coverage and R<r, i.e., the coverage overlapping region of the *n* nodes exists.

Next, we establish the general approach for finding the “last-connection” distance and the coordinate of its corresponding connection point. We only discuss the situations where the nodes are equipped with the unbounded convex model as an example. For the truncated convex models and concave models, the methods are similar to the unbounded models in the overlapping region centered at O(0,0), and the same with their corresponding 2-node cases [1] out of that overlapping region.

**Figure 2:**
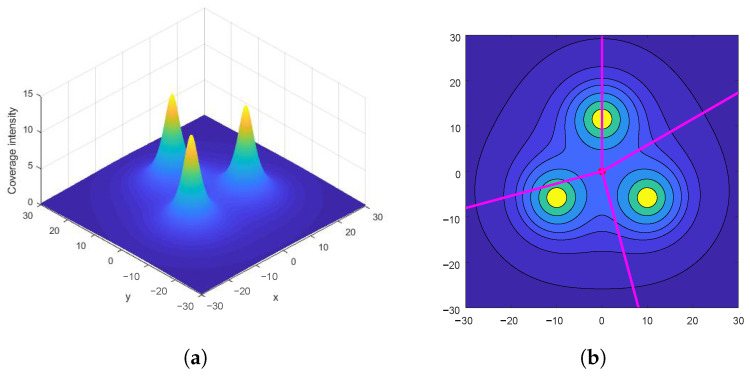
Coverage intensity of a 3-node team forming an equilateral triangle, with the nodes being located on the vertices. (**a**) Three-dimensional illustration, where the *z*-axis is the coverage intensity and the three peaks locate the three nodes; (**b**) contour plot with four arbitrary example rays.

**Figure 3:**
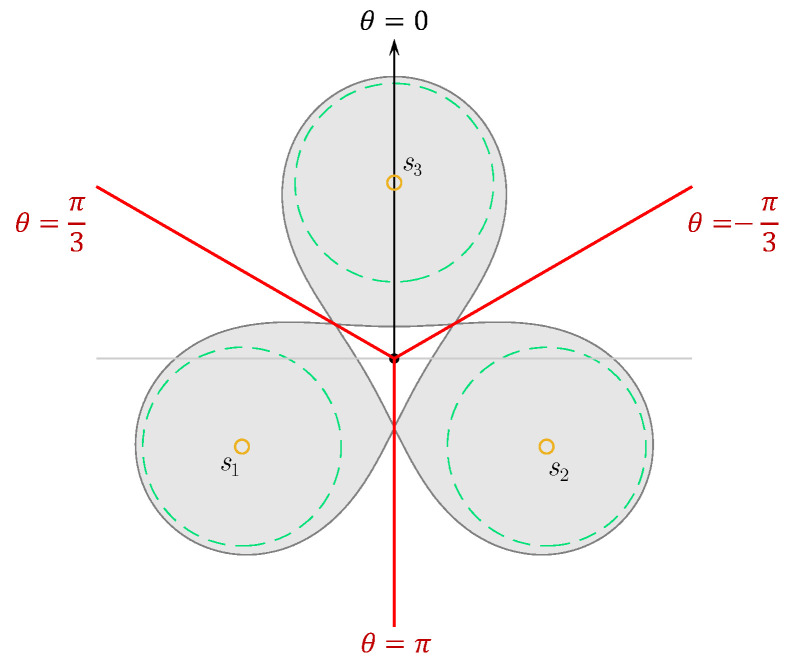
The “last-connection” points are on the mid-perpendicular line of every two adjacent nodes.

From the property of the convex decreasing functions, it is obvious that with any fixed θ value and ρ∈[0,+∞), Φ(p) has only two local extrema. The local minimum happens at O(0,0), which we already derived above. When ρ increases, Φ(p) increases up to its global maximum value and then decreases asymptotically to zero (see Figure 2). Based on symmetry, the “last-connection” points must be on the mirror line between every two nodes: their angle coordinates θ=π/3,π,−π/3, as shown in Figure 3. So, the ρ coordinate of these “last-connection” points can be found by determining the global maximum point of Φ(p) on any of the three rays θ=π/3,π,−π/3. Let’s look at the “last-connection” point $q_{1,2}\left(\rho ,\pi \right)$ on the ray θ=π (between $s_{1}$ and $s_{2}$), where the coverage intensity and its first-order derivative w.r.t. ρ are:

(7) $$\Phi \left(q_{1,2}\right)=2I\left(\sqrt{R^{2}+\rho^{2}-R\rho }\right)+I\left(R+\rho \right)$$

(8) $$\frac{\partial \Phi (q_{1,2})}{\partial \rho }={\left.\frac{dI}{dd}\right|}_{d=\sqrt{R^{2}+\rho^{2}-R\rho }}\left(\frac{2\rho -R}{\sqrt{R^{2}+\rho^{2}-R\rho }}\right)+{\left.\frac{dI}{dd}\right|}_{d=R+\rho }$$

The “last-connection distance” ($D=\sqrt{3}R$) and the ρ coordinate of its corresponding “last-connection” point can be found through solving the following equations:

(9) $$\left\{\begin{array}{c} \Phi (q_{1,2})=\gamma \\ \frac{\partial \Phi (q_{1,2})}{\partial \rho }=0\\ \rho >0 \end{array}\right.$$

An example analysis will be provided in Section 3.

### 2.2. General *n*-Node Team

After analyzing the effective coverage of 3-node teams, we now generalize it to *n*-node teams in *n*-sided polygon formations. We apply the usual notion of topological simple connectedness to characterize the effective coverage of a team. Consider the effectively covered region of team *S*. If there exist one or more “holes” within this region that are not effectively covered by *S*, then we say that the effectively covered region of *S* is not simply connected. Next, we establish a theory that enables us to easily examine whether the effectively covered region of the team equipped with an unbounded convex model of coverage is simply connected, i.e., whether any hole (that is not effectively covered) exists as a subregion within the effectively covered region.

**Theorem** **1.** *Weakest Point Theorem.*

*For an n-node team $S=\{s_{1},s_{2},\ldots ,s_{n}\}$ in the formation of an n-sided regular polygon where each node is equipped with a convex model of coverage and n≥3, its circumcenter O is effectively covered if and only if the entire effectively covered region is simply connected.*

**Proof.**  The sufficient condition is obvious. We only prove the necessary condition, which is equivalent to proving that the circumcenter O(0,0) is the only local minimum of coverage intensity Φ along any radial direction of the polar coordinate system (i.e., for any fixed θ value).

The first-order partial derivatives of *I* w.r.t. ρ and θ are

(10) $$\left\{\begin{array}{c} \frac{\partial I(d_{i})}{\partial \rho }=\frac{dI(d_{i})}{dd}\frac{\partial d}{\partial \rho }=\frac{dI(d_{i})}{dd}\frac{\rho -R\cos\left(\theta -\frac{2\pi i}{n}\right)}{\sqrt{R^{2}+\rho^{2}-2R\rho \cos\left(\theta -\frac{2\pi i}{n}\right)}}\\ \frac{\partial I(d_{i})}{\partial \theta }=\frac{dI(d_{i})}{dd}\frac{\partial d}{\partial \theta }=\frac{dI(d_{i})}{dd}\frac{R\rho \sin\left(\theta -\frac{2\pi i}{n}\right)}{\sqrt{R^{2}+\rho^{2}-2R\rho \cos\left(\theta -\frac{2\pi i}{n}\right)}} \end{array}\right.$$

When ρ=0, i.e., at the circumcenter O(0,0),

(11) $${\left.\frac{\partial \Phi (p)}{\partial \rho }\right|}_{\rho =0}=\sum_{i=1}^{n}\frac{\partial I(d_{i})}{\partial \rho }=-{\left.\frac{dI(d)}{dd}\right|}_{d=R}\sum_{i=1}^{n}\cos\left(\theta -\frac{2\pi i}{n}\right)=0$$

for any θ. See Appendix A for proof.

Then, the second-order partial derivative w.r.t. ρ is

(12) $$\begin{array}{cc} \frac{\partial^{2}I\left(d_{i}\right)}{\partial \rho^{2}} & =\frac{\frac{dI(d_{i})}{dd}}{\partial \rho }\frac{\partial d}{\partial \rho }+\frac{dI(d_{i})}{dd}\frac{\partial^{2}d}{\partial \rho^{2}}\\ \; & =\frac{d^{2}I\left(d_{i}\right)}{dd^{2}}\frac{{\left[\rho -R\cos\left(\theta -\frac{2\pi i}{n}\right)\right]}^{2}}{R^{2}+\rho^{2}-2R\rho \cos\left(\theta -\frac{2\pi i}{n}\right)}+\frac{dI(d_{i})}{dd}\frac{R^{2}\sin^{2}\left(\theta -\frac{2\pi i}{n}\right)}{{\left[R^{2}+\rho^{2}-2R\rho \cos\left(\theta -\frac{2\pi i}{n}\right)\right]}^{1.5}}. \end{array}$$

So, at the circumcenter *O*,

(13) $$\begin{array}{cc} {\left.\frac{\partial^{2}\Phi \left(p\right)}{\partial \rho^{2}}\right|}_{\rho =0} & ={\left.\frac{d^{2}I\left(d\right)}{dd^{2}}\right|}_{d=R}\sum_{i=1}^{n}\cos^{2}\left(\theta -\frac{2\pi i}{n}\right)+{\left.\frac{dI(d)}{dd}\right|}_{d=R}\frac{1}{R}\sum_{i=1}^{n}\sin^{2}\left(\theta -\frac{2\pi i}{n}\right)\\ \; & ={\left.\frac{d^{2}I\left(d\right)}{dd^{2}}\right|}_{d=R}\sum_{i=1}^{n}\frac{1+\cos\left(2\theta -\frac{4\pi i}{n}\right)}{2}+{\left.\frac{dI(d)}{dd}\right|}_{d=R}\frac{1}{R}\sum_{i=1}^{n}\frac{1-\cos\left(2\theta -\frac{4\pi i}{n}\right)}{2}\\ \; & ={\left.\frac{d^{2}I\left(d\right)}{dd^{2}}\right|}_{d=R}\frac{n}{2}+{\left.\frac{dI(d)}{dd}\right|}_{d=R}\frac{n}{2R}\\ \; & =\frac{n}{2}\left[{\left.\frac{d^{2}I\left(d\right)}{dd^{2}}\right|}_{d=R}+\frac{1}{R}{\left.\frac{dI(d)}{dd}\right|}_{d=R}\right]\\ \; & >0 \end{array}$$

Therefore, the circumcenter *O* is the local minimum point of the coverage intensity.

On the other hand obviously, when I(d) is concave and $d_{i}<r$, Equation (12) is always negative. Therefore,

(14) $$\frac{\partial^{2}\Phi \left(p\right)}{\partial \rho^{2}}=\sum_{i=1}^{n}\frac{\partial^{2}I\left(d_{i}\right)}{\partial \rho^{2}}<0\;\mathrm{when}\;R<r$$

which reveals that the coverage intensity Φ(O) at the circumcenter is the local maximum value in the coverage overlapping region of the *n* nodes equipped with a concave model. □

For a team where each node is equipped with an unbounded convex model of coverage, Theorem 1 implies that the circumcenter of the polygon formation is the weakest point in terms of the intensity throughout the team’s effectively covered region. When the separation distance *D* (side length of the polygon) increases, the effective coverage hole will appear at the circumcenter. The critical separation can be simply determined from $D=2I^{-1}\left(\gamma /n\right)\sin\left(\pi /n\right)$. Besides determining the hole existence of the effectively covered region of *S*, with Theorem 1, we can also determine the “last-connection distance” and its corresponding “last-connection” points by solving Equation (9), for this *n*-node team.

Although the connectedness of the effectively covered region is clearly analyzed, it is still impossible to find the explicit relation between team formation and the maximum team’s effective coverage. Some examples with explicit I(d) expressions will be derived, simulated, and compared in the Simulations section below.

## 3. Simulations

For 3-node teams, unbounded convex models and concave models are discussed, and the teams are in equilateral triangle formations. For *n*-node teams where n≥4, both regular polygon and regular star formations are involved, and equilateral triangular tessellation is simulated for the 6-node team. Optimal separations that maximize the corresponding effective coverages are found. Example plots of intensity vs. separation are presented. All simulations are performed in Matlab R2022a on Linux (Debian 10) OS, Intel Core i9-12900KF CPU.

### 3.1. Unbounded Convex Model for a 3-Node Team in Equilateral Triangle Formation

Let’s analyze the most common convex model

(15) $$I\left(d\right)=\frac{\beta }{d^{\lambda }+\alpha }$$

where the scaling factor and the exponent $\beta ,\lambda \in R^{+}$, and the offset constant α≥0. Its first-order derivative is

(16) $$\frac{dI}{dd}=-\frac{\beta \lambda d^{\lambda -1}}{{\left(d^{\lambda }+\alpha \right)}^{2}}$$

and the individual effective coverage radius is $\bar{r}=\sqrt[{\lambda }]{\beta /\gamma -\alpha }$. Substitute them into Equation (9):

(17) $$\begin{array}{cc} \frac{d\Phi (q_{1,2})}{d\rho }= & {\left.\frac{dI}{dd}\right|}_{d=\sqrt{R^{2}+\rho^{2}-R\rho }}\left(\frac{2\rho -R}{\sqrt{R^{2}+\rho^{2}-R\rho }}\right)+{\left.\frac{dI}{dd}\right|}_{d=R+\rho }\\ = & -\frac{\beta \lambda {\left(R^{2}+\rho^{2}-R\rho \right)}^{\frac{\lambda -1}{2}}}{{\left({\left(R^{2}+\rho^{2}-R\rho \right)}^{\frac{\lambda }{2}}+\alpha \right)}^{2}}\frac{2\rho -R}{\sqrt{R^{2}+\rho^{2}-R\rho }}-\frac{\beta \lambda {\left(R+\rho \right)}^{\lambda -1}}{{\left({\left(R+\rho \right)}^{\lambda }+\alpha \right)}^{2}}\\ = & -\beta \lambda \left[\frac{{\left(R^{2}+\rho^{2}-R\rho \right)}^{\frac{\lambda }{2}-1}}{{\left({\left(R^{2}+\rho^{2}-R\rho \right)}^{\frac{\lambda }{2}}+\alpha \right)}^{2}}\left(2\rho -R\right)+\frac{{\left(R+\rho \right)}^{\lambda -1}}{{\left({\left(R+\rho \right)}^{\lambda }+\alpha \right)}^{2}}\right]=0 \end{array}$$

Since β≠0 and λ≠0, we obtain

(18) $$\left\{\begin{array}{c} \frac{2}{{\left(R^{2}+\rho^{2}-R\rho \right)}^{\lambda /2}+\alpha }+\frac{1}{{\left(R+\rho \right)}^{\lambda }+\alpha }=\frac{1}{{\bar{r}}^{\lambda }+\alpha }\\ \frac{{\left(R^{2}+\rho^{2}-R\rho \right)}^{\frac{\lambda }{2}-1}}{{\left({\left(R^{2}+\rho^{2}-R\rho \right)}^{\frac{\lambda }{2}}+\alpha \right)}^{2}}\left(2\rho -R\right)+\frac{{\left(R+\rho \right)}^{\lambda -1}}{{\left({\left(R+\rho \right)}^{\lambda }+\alpha \right)}^{2}}=0\\ \rho >0 \end{array}\right.$$

This is a set of hyper equations when λ≠2 or α≠0. To make the equations solvable without losing universality, we set λ=2 and α=0, and the equations above become

(19) $$\left\{\begin{array}{c} \frac{2}{R^{2}+\rho^{2}-R\rho +0}+\frac{1}{R^{2}+\rho^{2}+2R\rho +0}=\frac{1}{{\bar{r}}^{2}+0}\\ \frac{2\rho -R}{{\left(R^{2}+\rho^{2}-R\rho +0\right)}^{2}}+\frac{R+\rho }{{\left({\left(R+\rho \right)}^{2}+0\right)}^{2}}=0\\ \rho >0 \end{array}\right.$$

After some algebras, we obtain

(20) $$\left\{\begin{array}{c} R^{4}+\rho R^{3}-3{\bar{r}}^{2}R^{2}+\left(\rho^{2}-3{\bar{r}}^{2}\right)\rho R+\rho^{4}-3{\bar{r}}^{2}\rho^{2}=0\\ \rho \left(\rho^{3}+R\rho^{2}+2R^{2}\rho -R^{3}\right)=0\\ \rho >0 \end{array}\right.$$

The two real roots of the second equation are ρ=0 and 0.3926R. Substitute ρ=0.3926R back to the first equation. Since R≠0, we get

(21) $$1.4770R^{2}-4.6405{\bar{r}}^{2}=0$$

Hence,

(22) $$R=1.7725\bar{r}$$

Therefore, for a 3-node team in equilateral triangle formation, the effectively covered region’s “last-connection” distance between the nodes (i.e., the side length of this equilateral triangle) happens at $D=\sqrt{3}R=3.0701\bar{r}$. At this critical separation distance, the whole region is only connected at three points $\left(0.6960\bar{r},\pi /3\right)$, $\left(0.6960\bar{r},\pi \right)$, $\left(0.6960\bar{r},-\pi /3\right)$. Any distance farther than this will result in the team’s effectively covered region being disconnected into three unconnected parts.

### 3.2. Concave Model for a 3-Node Team in Equilateral Triangle Formation

Many sensors follow concave models within their near field (short distances from the sensor). We use the quadratic difference equation to approximate the individual model of coverage:

(23) $$I\left(d\right)=\left\{\begin{array}{ccc} \alpha \left(r^{2}-d^{2}\right) & , & d\leq r\\ 0 & , & d>r \end{array}\right.$$

where $\alpha \in R^{+}$. The superposed coverage intensity in the overlapping region of the three nodes is

(24) $$\Phi \left(p\left(x,y\right)\right)=\alpha \left(3r^{2}-3x^{2}-3y^{2}-D^{2}\right)$$

Let Φ(p(x,y))≥γ; with individual effective coverage radius ${\bar{r}}^{2}=r^{2}-\gamma /\alpha$, we obtain the increased effective coverage region boundary in the overlapping region of the three nodes as

(25) $$x^{2}+y^{2}\leq \frac{2r^{2}+{\bar{r}}^{2}-D^{2}}{3}$$

when R<r, which is still a disk or part of a disk region.

**Figure 4:**
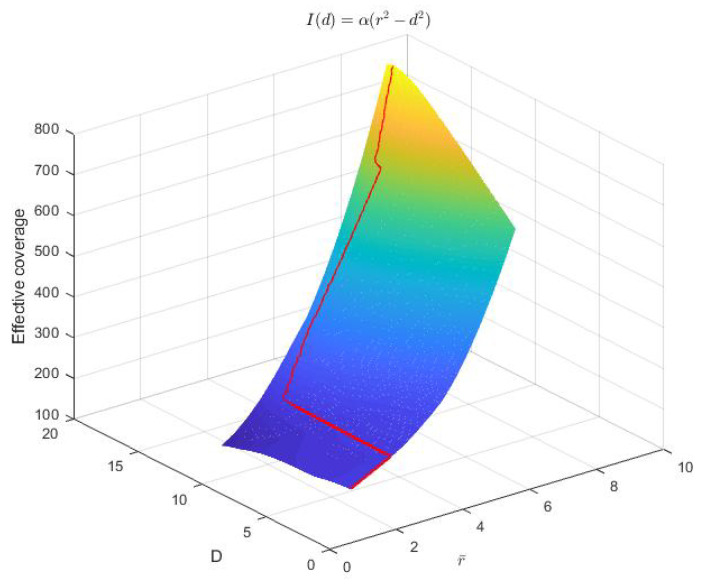
Three-dimensional mesh of team’s effective coverage vs. separation *D* and $\bar{r}$. Nodes are equipped with concave models. r=10. α=1. The red curve illustrates the maximum effective coverage at every $\bar{r}$ value.

**Figure 5:**
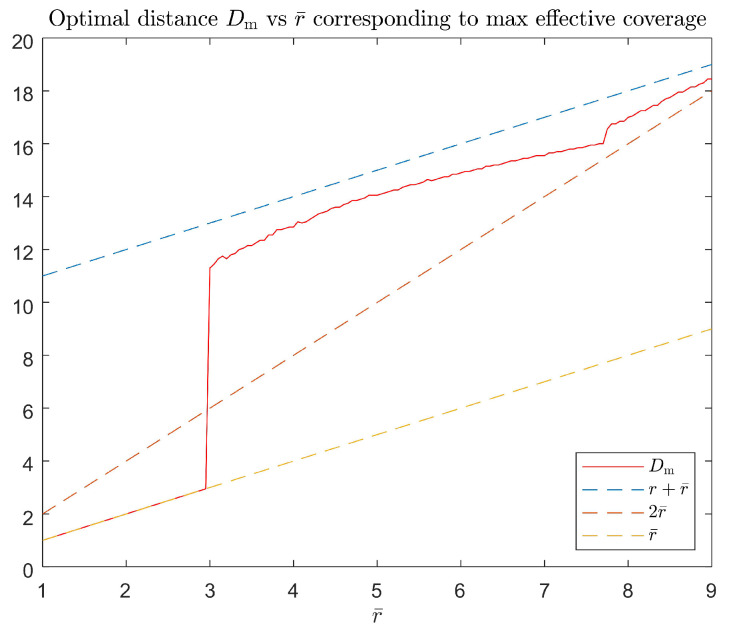
Relation between the optimal *D* and $\bar{r}$ corresponding to the maximum team’s effective coverage.

**Figure 6:**
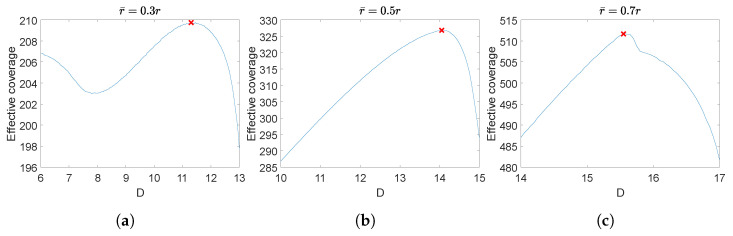
Team’s effective coverage vs. separation *D* under three different thresholds: (**a**) $\bar{r}=0.3r$, (**b**) $\bar{r}=0.5r$, (**c**) $\bar{r}=0.7r$. Nodes are equipped with concave models. r=10. α=1. The red cross in each plot marks the maximum effective coverage.

**Figure 7:**
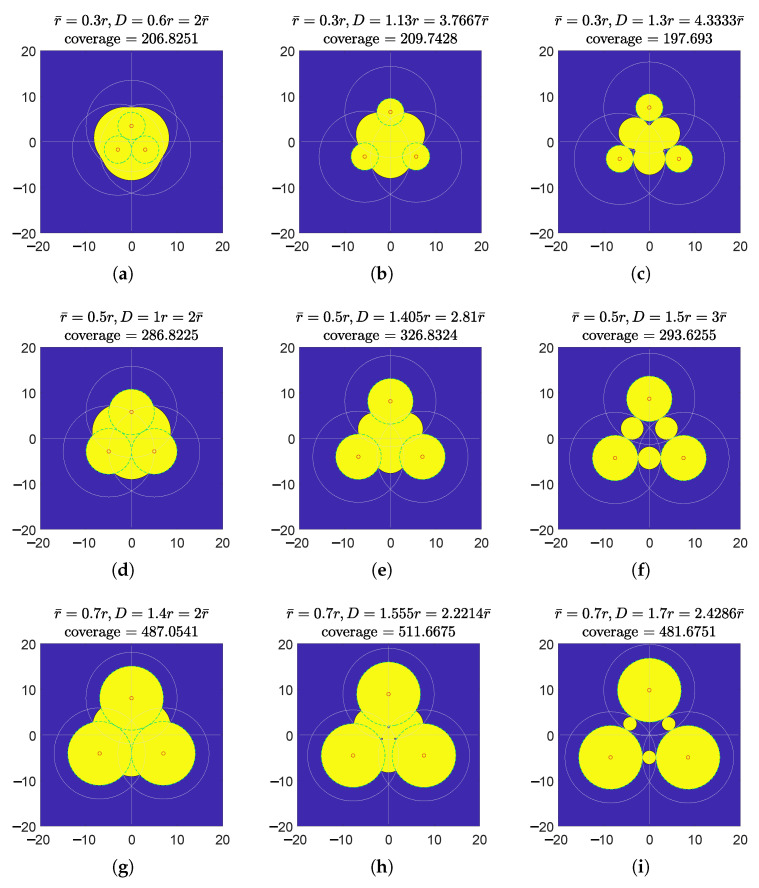
Illustration of resultant effective coverages of the 3-node team equipped with concave models $I\left(d\right)=\alpha \left(r^{2}-d^{2}\right)$ under different thresholds γ (intuitively reflected as $\bar{r}$). Each row represents one value of γ: they are $\bar{r}=0.3r,0.5r,0.7r$, respectively. Each column represents a different separation distance *D*: the first column (**a**,**d**,**g**) is the “tangent column”, where the individual effectively covered regions in light green dashed circles are tangent to each other, i.e., $D=2\bar{r}$; the second column (**b**,**e**,**h**) is the “max column”, where the team’s effective coverage is maximized with corresponding separations *D*; the third column (**c**,**f**,**i**) shows configurations where $D=r+\bar{r}$. r=10. α=1.

Next, we simulate this model with different *D* and $\bar{r}$ values. The scaling factor α=1 and the individual coverage radius r=10. $\bar{r}=0.1r$ to 0.9r and $D=2\bar{r}$ to $\bar{r}+r$ are examined, whose team’s effective coverage is meshed in Figure 4. The optimal separation $D_{m}$ vs. these $\bar{r}$ values are plotted in Figure 5. The effective coverages vs. *D* under three thresholds $\bar{r}=0.3r,0.5r,0.7r$ are selected to be plotted in Figure 6, and their effectively covered regions are shown in Figure 7.

As we can see from Figure 7, the maximum effective coverage may or may not have effective coverage hole(s). In the case that the threshold γ is low (resulting in large $\bar{r}$), it will have holes. When separation *D* increases, three effective coverage holes first appear at the three corners of the 3-node overlapping region, and then merge into one hole; after the effectively covered region in the center overlapping region disappears, the entire effectively covered region becomes simply the combination of the 2-node case.

**Figure 8:**
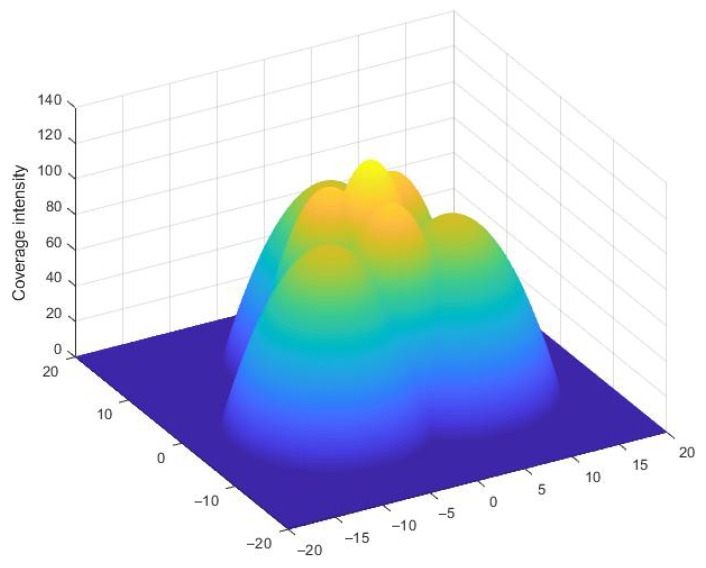
Three-dimensional illustration of coverage intensity of the 3-node team equipped with a concave model of coverage, in an equilateral triangle formation, with the nodes located on its vertices. The *z*-axis is the coverage intensity. r=10. α=1.

From Figure 4 and Figure 5, it is clearly seen that when the threshold γ is high ($\bar{r}$ is small), the optimal separation is $\bar{r}$; when $\bar{r}$ is roughly larger than 0.3r, the optimal separation $D_{m}$ falls between $2\bar{r}$ and $r+\bar{r}$. Figure 8 shows that the coverage intensity has seven local maxima located at the three nodes, three midpoints between every two nodes, and the circumcenter.

### 3.3. n Nodes in Regular Polygon and Regular Star Formations

For *n*-node teams where n≥4, *n*-sided regular polygons and (n−1)-vertex regular star formations, and the 6-node team in equilateral triangular tessellation formation are simulated and compared. The term “(n−1)-vertex regular star” refers to the formation of an (n−1)-sided regular polygon with another node placed at its circumcenter. Each node is equipped with the unbounded convex model $I\left(d\right)=\beta /d^{2}$, where $\beta \in R^{+}$. In this subsection, the scaling factor β=1.

**Figure 9:**
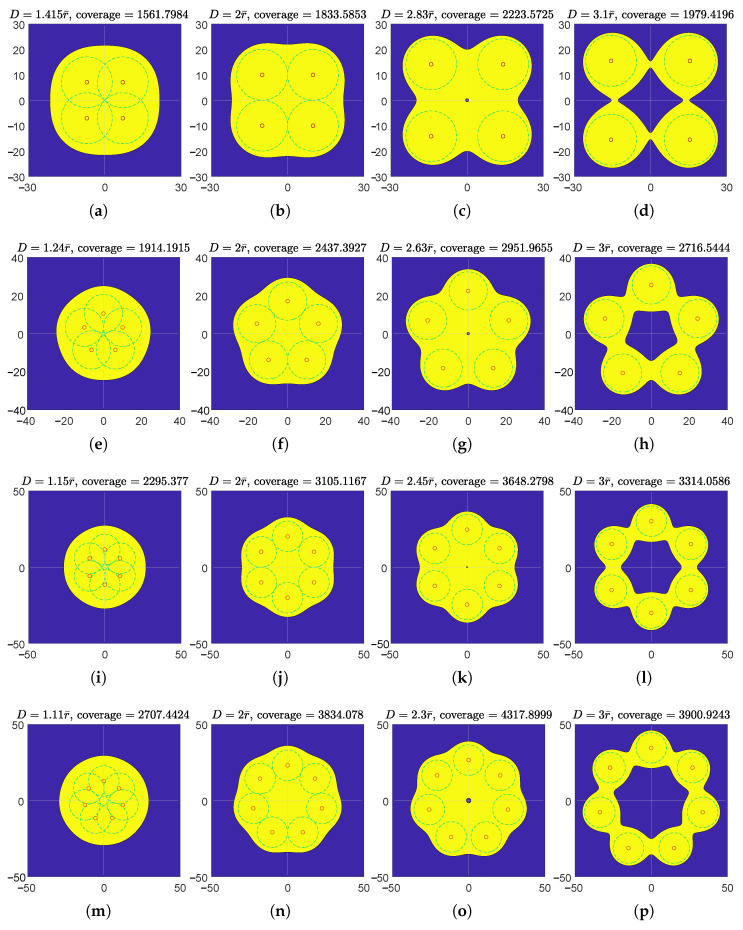
Illustration of effective coverages of *n*-node teams equipped with model $I\left(d\right)=\beta /d^{2}$ where n=4,5,6,7. The teams are in regular polygon formations. $\bar{r}=10$. Each row represents an *n* value. In the first column (**a**,**e**,**i**,**m**), individual effective coverage disks of every other node (i.e., *i* and i+2) on the polygon vertices are tangent. In the second column (**b**,**f**,**j**,**n**), the individual effective coverage disks of every adjacent node pair are tangent. The third column (**c**,**g**,**k**,**o**) is the “max column”, where teams’ effective coverages are maximized. Each effectively covered region in the fourth column (**d**,**h**,**l**,**p**) has an effective coverage hole inside, while the region is still connected.

**Figure 10:**
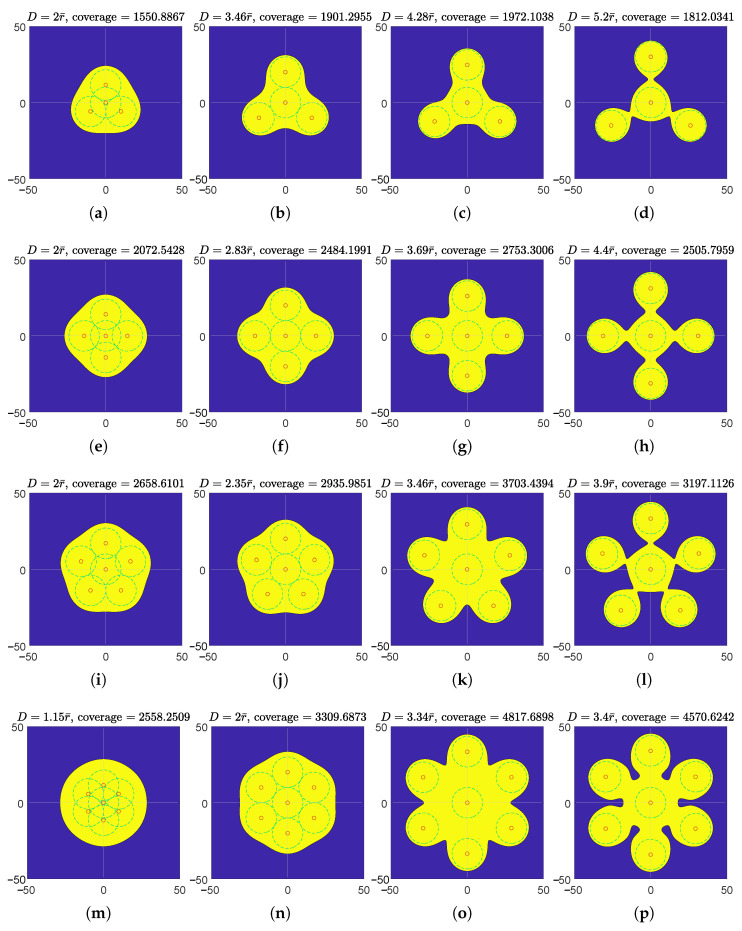
Illustration of effective coverages of *n*-node teams equipped with model $I\left(d\right)=\beta /d^{2}$, where n=4,5,6,7. The teams are in regular star formations. $\bar{r}=10$. Each row represents an *n* value. In the first column, individual effective coverage disks of every adjacent node pair are tangent in (**a**,**e**,**i**), and individual effective coverage disks of every other node on the polygon vertices are tangent in (**m**). In the second column (**b**,**f**,**j**,**n**), the individual effective coverage disks of nodes on the vertices are tangent to that of the center node. The third column (**c**,**g**,**k**,**o**) is the “max column”, where teams’ effective coverages are maximized. The fourth column (**d**,**h**,**l**,**p**) shows the effective coverages at randomly picked separations while the region is still connected.

**Figure 11:**
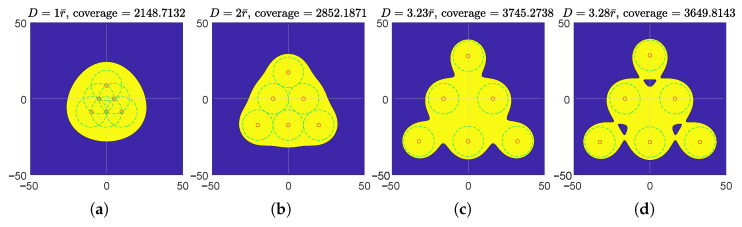
Illustration of effective coverages of *n*-node teams equipped with model $I\left(d\right)=\beta /d^{2}$, where n=6. The teams are in equilateral triangle formations. $\bar{r}=10$. (**a**) $D=\bar{r}$; (**b**) $D=2\bar{r}$, individual effective coverage disks of adjacent nodes are tangent with each other; (**c**) the team’s effective coverage maximized; (**d**) the effective coverage at randomly picked separation while the region is still connected.

The effectively covered regions of 4,5,6,7-node teams in the polygon and star formations are illustrated in Figure 9 and Figure 10, respectively. In both the regular polygon and regular star formations, the maximum teams’ effective coverages are achieved when the individual effective coverage disks are apart from each other (neither tangent nor secant), but the entire effectively covered region is still connected. In regular polygon formations, an effective coverage hole is observed in each maximum effective coverage illustration at the circumcenter, which also verified in Theorem 1. Additionally, the effectively covered regions of the six-node team in equilateral triangle formation are illustrated in Figure 11. Its maximum effective coverage is found to be slightly larger than the previous two formations, with n=6.

**Figure 12:**
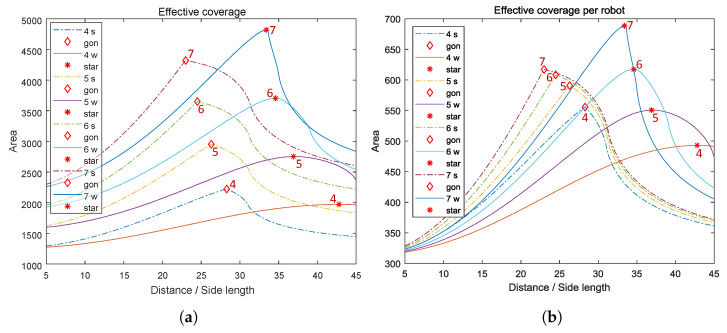

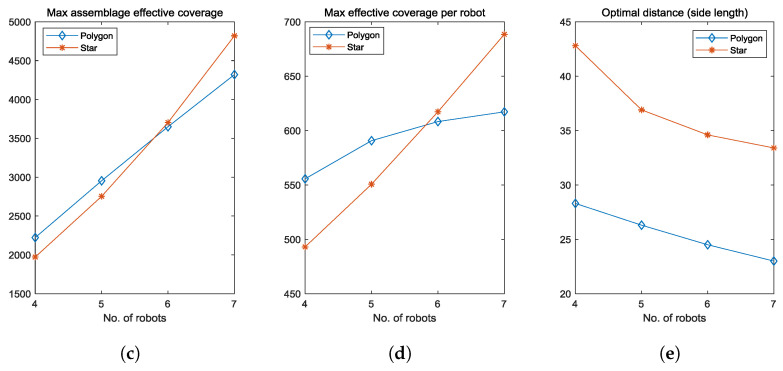
Plots of effective coverages of *n*-node teams equipped with model $I\left(d\right)=\beta /d^{2}$, where n=4,5,6,7. The team formations are in regular polygons (⋄) and regular stars (*). $\bar{r}=10$. (**a**) Team’s effective coverage; (**b**) Effective coverage per node; (**c**) Maximum team’s effective coverage vs. the number of nodes; (**d**) Maximum effective coverage per node vs. the number of nodes; (**e**) Optimal distance (polygon side length) vs. the number of nodes.

The effective coverages of a team and per node are plotted in Figure 12. From Figure 12a, it is seen that the team’s effective coverage naturally increases with the number of nodes in both formations. It can be observed that with n=4,5, regular polygon formations generate larger maximum effective coverages than regular star formations; while with larger *n* values (n=6,7), the regular star formations instead generate larger maximum effective coverages than regular polygon formations. This trend is further revealed in subplot (c). From Figure 12b, it is found that for both regular polygon and regular star formations, the maximum effective coverage per node increases with the number of nodes *n*. This is further revealed in subplot (d), where we can see that the single-node contribution to effective coverage tends to increase slower when the number of nodes *n* increases. The optimal side lengths of both polygon and star formations decrease as the number of nodes *n* grows, which is shown in Figure 12e.

### 3.4. Comparison

To justify the effectiveness of our work in solving the given problem, a comparison with the existing binary model and the non-constant models in infinite planes (e.g., [5]) is conducted. By applying Equation (15) as the real sensing quality (confidence) function, the team’s cooperative sensing quality at a point *p* is $\Phi \left(p\right)=1-\prod_{i=1}^{n}\left(1-I(d_{i})\right)=1-\prod_{i=1}^{n}\left(1-\frac{\alpha }{d_{i}^{\lambda }+\alpha }\right)$. Let’s examine n=4 as an example. By using the same method with previous subsections, the optimal side length $D_{m}$ and the largest effective coverage of the team are obtained and logged in Table 1. With the binary model ($\bar{r}=r$), the team in regular polygon formation achieves the maximum effective coverage at D≥2r obviously; while to keep its connectedness, its $D_{m}$ has to be 2r.

From Table 1, it can be easily seen that our model obtains the closest optimal formations and their corresponding effective coverage values with the ground truth. This is because the mathematical expression of the binary model is too far from that of a real device. The models for solving the coverage problem of an infinite plane only consider the portion inside a polygon lattice of the infinite plane; the rest of the portion outside of the polygon lattice is considered as simple repetitions in other neighboring polygons, thus causing larger errors in approximation.

## 4. Conclusions

In this article, the general properties of the effective coverage of an *n*-node team (n≥3) equipped with radial attenuation disk models following the superposition principle have been studied. For the 3-node team equipped with an unbounded convex model in the equilateral triangle formation, the general approach for finding the “last-connection” distance and its corresponding point coordinates are established. Example analyses and numerical simulations on both the unbounded convex model and the concave model have been conducted. For an *n*-node team (n≥4) in regular polygon formation where nodes are equipped with an unbounded convex model, it is proven that the circumcenter of the formation is the point with the weakest coverage intensity in the team’s effectively covered region. Formations of regular polygons, regular stars, and an equilateral triangular tessellation (for six nodes) are simulated, through which the optimal formations are found. From the numerical result, for the 3-node team equipped with a concave model, the optimal distance is the individual effective coverage radius $\bar{r}$ when the threshold is high ($\bar{r}$ is small), and it falls between two times the individual effective coverage radius, the sum of the individual coverage radius, and the individual effective coverage radius ($r+\bar{r}$) when the individual effective coverage radius is roughly larger than 0.3 times the individual coverage radius. For 4- and 5-node teams, regular polygon formations have larger maximum effective coverages than their corresponding regular star formations, while when the number of nodes is larger than 6, the situation is just the opposite. For a 6-node team equipped with an unbounded convex model, the equilateral triangle formation is the optimal one with the largest maximum effective coverage among the three formations. For both the regular polygon and the regular star formations, the more the cooperative nodes, the larger the maximum effective coverage per node. The single-node contribution to the effective coverage of the team increases more slowly when the number of nodes increases. It is seen that the simulation results comply with the theoretical analysis and validate our propositions and theories. The comparison among our model, the binary model, and the method used for covering an infinite plane shows that our model is more accurate. This study provides useful insights and guides to the deployment of practical devices (e.g., sensors and other output devices) in coverage applications.

## Figures and Tables

**Table 1:** Comparison of $D_{m}$ and $\bar{C}$ values with n=4.

α	Ground Truth	Our Convex Model	Binary Model	Infinite Plane Model (e.g., [5])
1	$2.835\bar{r}$, 2229.882	$2.84\bar{r}$, 2235.9219	$2\bar{r}$, 1256.6371	$2.845\bar{r}$, 3222.7623
3	$2.845\bar{r}$, 2241.6723	$2.86\bar{r}$, 2259.9843	$2\bar{r}$, 1256.6371	$2.87\bar{r}$, 3271.84
5	$2.855\bar{r}$, 2253.7023	$2.88\bar{r}$, 2283.6948	$2\bar{r}$, 1256.6371	$2.89\bar{r}$, 3317.76
7	$2.865\bar{r}$, 2265.4527	$2.9\bar{r}$, 2307.0858	$2\bar{r}$, 1256.6371	$2.915\bar{r}$, 3364
10	$2.88\bar{r}$, 2282.5741	$2.93\bar{r}$, 2342.5067	$2\bar{r}$, 1256.6371	$2.945\bar{r}$, 3436.362

**Figure A1:**
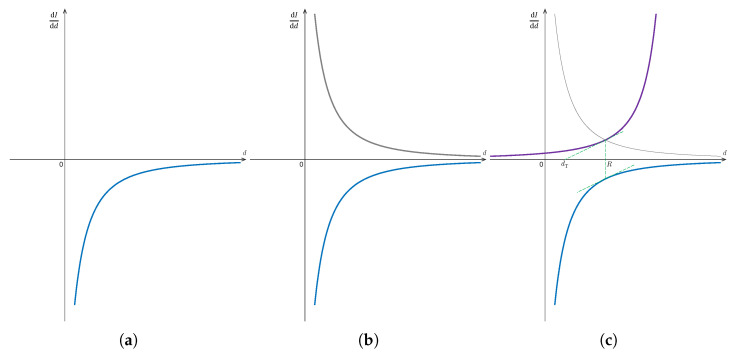
Plots of the first-order derivative of a convex model of coverage and its mirrors: (**a**) original first-order derivative of the convex model (blue), (**b**) mirrored about *d* axis (gray), and (**c**) further mirrored about d=R (violet).

## Data Availability

No extra data other than those reported in the article is available.

## Abbreviations

The following abbreviations are used in this manuscript:

WSN   Wireless Sensor Network

## Appendix A. Sum of a Sine Function with Equidistant Phases

The following equation holds for $\theta \in R,n\in Z^{+},n\geq 2$:

$$\sum_{i=1}^{n}\cos\left(\theta -\frac{2\pi i}{n}\right)=0$$

There are three ways to prove this:

### Appendix A.1. Proof by Contradiction Using a Vector Rotation Approach

Let’s construct a group of *n* vectors $\cos\left(\theta -\frac{2\pi i}{n}\right)x+\sin\left(\theta -\frac{2\pi i}{n}\right)y$ for i=1,2,⋯,n with a common initial point at the origin. Assume the sum vector

$$\sum_{i=1}^{n}\left[\cos\left(\theta -\frac{2\pi i}{n}\right)x+\sin\left(\theta -\frac{2\pi i}{n}\right)y\right]$$

is a non-zero vector. When the entire vector group is rotated about the origin for 2π/n, it becomes exactly the same as before rotation, but the non-zero sum vector is also rotated for 2π/n. Hence, to be equal before and after the rotation, the sum vector must be a zero vector, i.e., $\sum_{i=1}^{n}\cos\left(\theta -\frac{2\pi i}{n}\right)=\sum_{i=1}^{n}\sin\left(\theta -\frac{2\pi i}{n}\right)=0$ holds for any θ∈R.

### Appendix A.2. Vieta’s Formula Approach

We know that the *i*th root of equation $x^{n}=1$ is $\cos\frac{2\pi i}{n}+j\sin\frac{2\pi i}{n}$. From Vieta’s formulas of relations between roots and coefficients, we have

(A1) $$\sum_{i=1}^{n}\left(\cos\frac{2\pi i}{n}+j\sin\frac{2\pi i}{n}\right)=0$$

which implies $\sum_{i=1}^{n}\cos\frac{2\pi i}{n}=\sum_{i=1}^{n}\sin\frac{2\pi i}{n}=0$. Hence,

(A2) $$\sum_{i=1}^{n}\cos\left(\theta -\frac{2\pi i}{n}\right)=\cos\theta \sum_{i=1}^{n}\cos\frac{2\pi i}{n}+\sin\theta \sum_{i=1}^{n}\sin\frac{2\pi i}{n}=0.$$

### Appendix A.3. Eular’s Formula Approach

From Eular’s formula, we have

(A3) $$\begin{array}{cc} \; & \sum_{i=1}^{n}\cos\left(\theta -\frac{2\pi i}{n}\right)+j\sum_{i=1}^{n}\sin\left(\theta -\frac{2\pi i}{n}\right)\\ = & \sum_{i=1}^{n}e^{j\left(\theta -\frac{2\pi i}{n}\right)}\\ = & e^{j\theta }\sum_{i=1}^{n}e^{-j\frac{2\pi i}{n}}\\ = & e^{j\left(\theta -\frac{2\pi }{n}\right)}\frac{1-e^{-i2\pi }}{1-e^{-j\frac{2\pi }{n}}}\\ = & e^{j\left(\theta -\frac{2\pi }{n}\right)}\frac{1-1}{1-e^{-j\frac{2\pi }{n}}}\\ = & 0. \end{array}$$

Hence, $\sum_{i=1}^{n}\cos\left(\theta -\frac{2\pi i}{n}\right)=\sum_{i=1}^{n}\sin\left(\theta -\frac{2\pi i}{n}\right)=0$ holds for any θ∈R.

## Appendix B. Relation between First- and Second-Order Derivatives of a Convex Decreasing Function

For a convex model of coverage I(d), $\forall R\in R^{+}$, the following inequality always holds:

(A4) $${\left.\frac{d^{2}I\left(d\right)}{dd^{2}}\right|}_{d=R}+\frac{1}{R}{\left.\frac{dI(d)}{dd}\right|}_{d=R}>0$$

**Proof.** Convex models of coverage possess the following properties:

- I(d)>0 on $d\in R^{+}$
- $\frac{dI(d)}{dd}<0$ on $d\in R^{+}$
- $\frac{d^{2}I\left(d\right)}{dd^{2}}>0$ on $d\in R^{+}$
- $\lim_{d\to +\infty }I\left(d\right)=0$
- $\lim_{d\to +\infty }\frac{dI(d)}{dd}=0$

According to the above properties, we approximately draw the curve for the first derivative dI/dd v.s. *d*, which entirely falls in the fourth quadrant and is asymptotic to *d*-axis, as shown in Figure A1a. Then, we flip the function image about the *d*-axis to obtain a new curve for −dI(d)/dd, as shown in Figure A1b. Next, we flip the new image again about the line d=R to obtain the final curve for −dI(2R−d)/dd, as shown in Figure A1c. On the final curve, the derivative at d=R equals that on the original curve:

(A5) $${\left.-\frac{d^{2}I\left(2R-d\right)}{dd^{2}}\right|}_{d=R}={\left.\frac{d^{2}I\left(d\right)}{dd^{2}}\right|}_{d=R}>0$$

We draw the tangent line on curve −dI(2R−d)/dd at d=R, which intersects *d*-axis at $d_{T}$, as shown in Figure A1. Since dI(d)/dd is concave, −dI(2R−d)/dd is convex, and $d_{T}>0$. So, we have

(A6) $$-{\left.\frac{dI(d)}{dd}\right|}_{d=R}={\left.-\frac{d^{2}I\left(2R-d\right)}{dd^{2}}\right|}_{d=R}\left(R-d_{T}\right)<{\left.\frac{d^{2}I\left(d\right)}{dd^{2}}\right|}_{d=R}R$$

Therefore,

(A7) $$\frac{1}{R}{\left.\frac{dI(d)}{dd}\right|}_{d=R}+{\left.\frac{d^{2}I\left(d\right)}{dd^{2}}\right|}_{d=R}>0$$

□
